# Women’s preferences for alternative financial incentive schemes for breastfeeding: A discrete choice experiment

**DOI:** 10.1371/journal.pone.0194231

**Published:** 2018-04-12

**Authors:** Frauke Becker, Nana Anokye, Esther W. de Bekker-Grob, Ailish Higgins, Clare Relton, Mark Strong, Julia Fox-Rushby

**Affiliations:** 1 Health Economics Research Centre, Nuffield Department of Population Health, University of Oxford, Oxford, United Kingdom; 2 Health Economics Research Group, Brunel University, Uxbridge, Middlesex, United Kingdom; 3 Section of Health Technology Assessment and Erasmus Choice Modelling Centre, Erasmus School of Health Policy & Management, Erasmus University Rotterdam, Rotterdam, The Netherlands; 4 School of Health and Related Research, University of Sheffield, Sheffield, United Kingdom; Christiana Care Health System, UNITED STATES

## Abstract

**Background:**

Increasing breastfeeding rates have been associated with reductions in disease in babies and mothers as well as in related costs. ‘Nourishing Start for Health (NoSH)’, a financial incentive scheme has been proposed as a potentially effective way to increase both the number of mothers breastfeeding and duration of breastfeeding.

**Aims:**

To establish women’s relative preferences for different aspects of a financial incentive scheme for breastfeeding and to identify importance of scheme characteristics on probability on participation in an incentive scheme.

**Methods:**

A discrete choice experiment (DCE) obtained information on alternative specifications of the NoSH scheme designed to promote continued breastfeeding duration until at least 6 weeks after birth. Four attributes framed alternative scheme designs: value of the incentive; minimum breastfeeding duration required to receive incentive; method of verifying breastfeeding; type of incentive. Three versions of the DCE questionnaire, each containing 8 different choice sets, provided 24 choice sets for analysis. The questionnaire was mailed to 2,531 women in the South Yorkshire Cohort (SYC) aged 16–45 years in IMD quintiles 3–5. The analytic approach considered conditional and mixed effects logistic models to account for preference heterogeneity that may be associated with a variation in effects mediated by respondents’ characteristics.

**Results:**

564 women completed the questionnaire and a response rate of 22% was achieved. Most of the included attributes were found to affect utility and therefore the probability to participate in the incentive scheme. Higher rewards were preferred, although the type of incentive significantly affected women’s preferences on average. We found evidence for preference heterogeneity based on individual characteristics that mediated preferences for an incentive scheme.Conclusions

Although participants’ opinion in our sample was mixed, financial incentives for breastfeeding may be an acceptable and effective instrument to change behaviour. However, individual characteristics could mediate the effect and should therefore be considered when developing and targeting future interventions.

## 1. Introduction

Breastfeeding promotes health and prevention of disease in both infant and mother in the short and long term [1, 2]. The World Health Organisation recommends exclusive breastfeeding for the first 6 months after birth [3]. However, breastfeeding rates in the UK are among the lowest worldwide where rates for continued breastfeeding decrease rapidly after birth [4], contributing to increased preventable illnesses and substantial associated health care costs [1, 5].

Pokhrel, Quigley [2] found that supporting mothers in breastfeeding exclusively for longer durations was associated with substantial cost savings, both from reduced breast cancer prevalence and health improvements related to four acute conditions in infants. In order to reach this cost saving potential, exclusive breastfeeding rates would have to increase from 7% to 45% at 4 months and from 35% to 75% at discharge from hospital. However, this study indicated the need for evidence on the costs and effects of specific interventions targeted at increasing breastfeeding rates.

Financial incentives have been proposed as an effective approach to promote health behaviours [6–9]. However, the impact of financial incentives on breastfeeding rates and durations in the UK in particular is still largely unexplored. The effectiveness and cost-effectiveness of an incentive scheme is currently being tested in the Nourishing Start for Health (NoSH) cluster-randomised trial [10] in order to inform future policy on breastfeeding.

Societal concerns regarding financial incentives for breastfeeding have been identified previously [11]. Although they are thought to encourage mothers to breastfeed, there is concern that financial incentives for breastfeeding could be “discriminatory and divisive” as well as “personally insulting”. Preferences among potential recipients for a financial incentive scheme remain unexplored.

Discrete choice experiments (DCEs) are a method to explore preferences where data on observational behaviour is not available. They are well established in health economics [12–14] and increasingly used in public health [15–17]. DCEs have been used to identify relative preferences for different characteristics of financial incentives aimed at changing health behaviours [6, 18, 19]. Findings indicate that the effectiveness of financial incentives depends on social acceptability of the specific health behaviour. More flexible payments (i.e. cash) are usually preferred to payments that are limited in their validity because they can only be used in a certain type of shop.

In order to help inform the design of the financial incentive scheme for the NoSH trial, we conducted a DCE exploring women’s relative preferences for characteristics of an incentive scheme [20–22]. A better understanding of individual preferences and societal acceptability would help to develop and increase the effectiveness of a future scheme.

## 2. Methods

Discrete choice experiments (DCEs) describe hypothetical interventions according to their key characteristics, or ‘attributes’ (e.g. type of reward, value of incentive), and ‘levels’ of these attributes (e.g. cash, shopping voucher). Participants are then asked which of the presented intervention scenarios, combining different levels of each attribute, they prefer, assuming that they choose the scenario that would result in the highest utility or lowest regret for them. This gives an indication for participants’ underlying preferences and allows relative preferences for attribute levels to be determined.

The methods below describe development of the postal questionnaire, experimental design for the DCE, source of data and analyses.

### 2.1. Development of postal questionnaire

Informed by a structured development process, we designed a self-completed questionnaire. It included sections on experience with infant feeding, ranking exercises for respondents’ preferences related to two out of the four attributes (type of incentive, method to verify breastfeeding) for validation purposes, household information (e.g. income, employment status) in order to be able to control for potential preference heterogeneity, and a DCE to explore women’s own preferences for a breastfeeding incentive scheme.

The design and application of the DCE comprised two stages:

- development and pre-testing of a self-completed questionnaire,
- postal survey.

The attributes and levels associated with financial incentives for breastfeeding were informed from literature review, focus group discussions and face to face interviews with women, health professionals and commissioners as well as extensive pre-testing among eligible women until data saturation was reached. Qualitative interviews during the pre-testing suggested that respondents understood the materials presented and were able to complete the questionnaire without assistance.

Table 1 sets out the attributes and levels used in the choice scenarios and displays the frequency each attribute level appeared in each of the questionnaire versions. Four attributes with associated levels were identified: minimum duration of breastfeeding required to receive the incentive payment, maximum total value of incentive payment, type of financial incentive, and method to verify breastfeeding. Levels for the duration of breastfeeding varied between 2 days, 10 days and 6 weeks. Amounts for the value of incentives were determined based on parts of the pre-testing that focussed only on payment levels. We used payment cards with pre-defined values as well as an open-ended question on the maximum amount that women felt should be paid as an incentive. The maximum total value included in the DCE scenarios was specified as either £20, £40, £80, £120, £240 or £600 per duration of breastfeeding described in the specific scenario. In previous studies [23, 24] financial incentives were given in form of cash, shopping vouchers or lottery tickets. Local decision makers in the study area were interested in vouchers for local shops as part of regeneration strategies. Based on the literature review and additional focus group work, types of financial incentives included in our questionnaire were direct cash transfer, vouchers for either high street or local shops, and a gift pack. The additional level of a gift pack was included since focus group work brought up the issue around mis-use of additional income in form of a financial incentive. The majority of interviewed women felt that any additional income should be spent on the mother or her baby, which could be guaranteed by a gift pack. Breastfeeding could be verified by signed statement(s) by either the mother, her health care provider, or both.

**Table 1:** Attributes, levels and level balance (24 choice sets with 2 hypothetical scheme options).

Attributes	Levels	Number of appearances			
		Total n	Version 1 n (%)	Version 2 n (%)	Version 3 n (%)
Duration of breastfeeding [days]	2	16	5 (31)	5 (31)	6 (38)
	10	16	7 (44)	4 (25)	5 (31)
	42	16	4 (25)	7 (44)	5 (31)
Max. amount [£]	20	8	4 (25)	2 (13)	2 (13)
	40	8	0 (0)	5 (31)	3 (19)
	80	8	3 (19)	2 (13)	3 (19)
	120	8	5 (31)	0 (0)	3 (19)
	240	8	2 (13)	3 (13)	3 (19)
	600	8	2 (13)	4 (25)	2 (13)
Type of financial incentive	Direct cash transfer	12	4 (25)	5 (31)	3 (19)
	Vouchers: high street shops	12	3 (19)	3 (19)	6 (38)
	Vouchers: local shops	12	4 (25)	5 (31)	3 (19)
	Gift pack	12	5 (31)	3 (19)	4 (25)
Method of confirmation: Signed statement	Mother	16	6 (38)	5 (31)	5 (31)
	Mother & health care provider	16	5 (31)	4 (25)	7 (44)
	Health care provider	16	5 (31)	7 (44)	4 (25)

**Fig 1:**
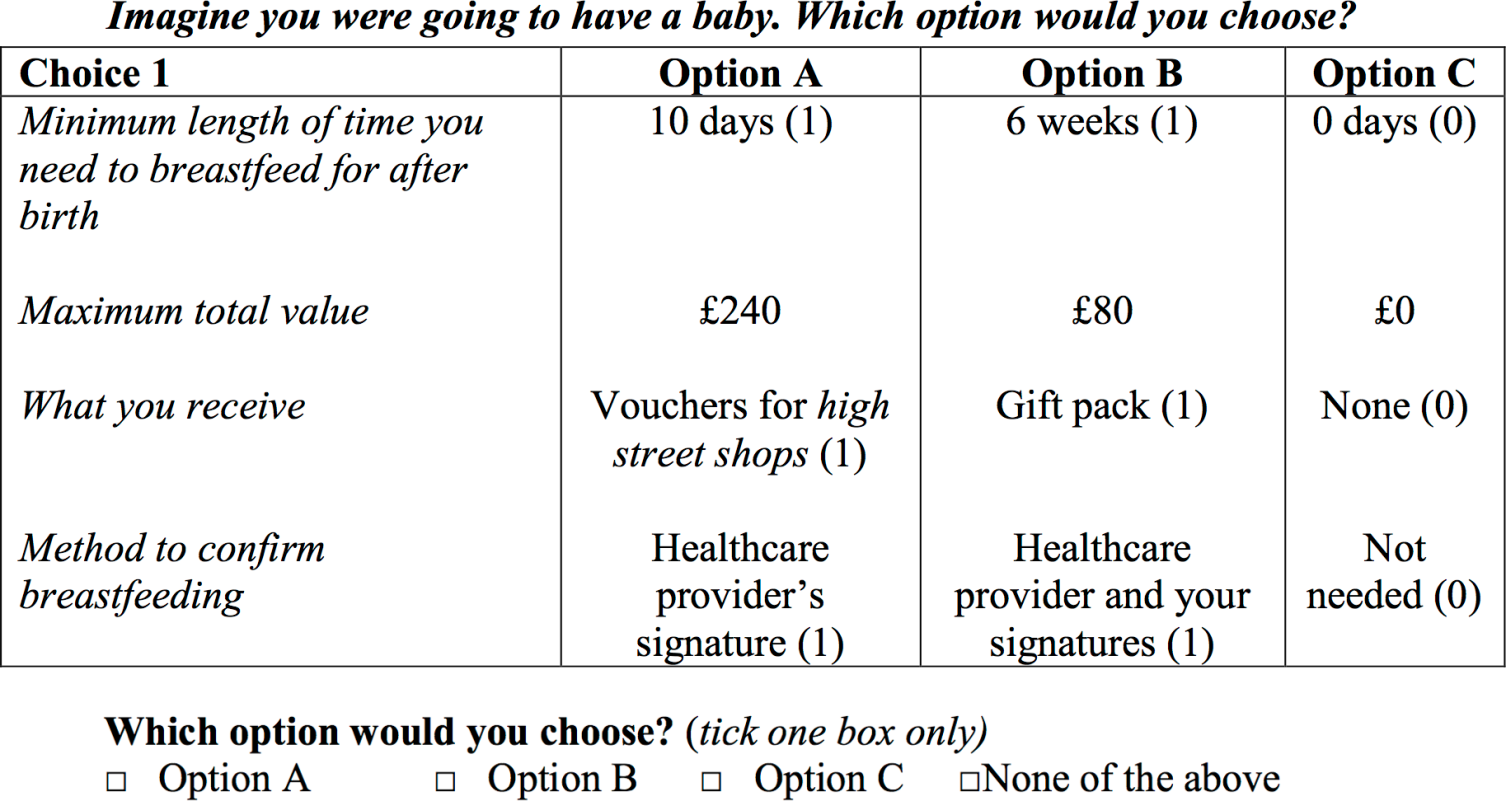
Example of a choice set used in the DCE (coding options for effects coding in brackets).

The four attributes and associated levels were combined in scenarios presented in choice sets from which respondents had to choose one option (see Fig 1 for a choice set example). The total number of possible scenarios was 216. Observations and feedback during pre-testing showed that participants found more than 8 choice sets to be too burdensome. They lost focus and would skim through the presented scenarios. Therefore in order to minimise the cognitive burden for respondents, each questionnaire included eight choice sets containing three alternative un-labelled scenarios (“Option A”, “Option B”, “Option C”) from which women were asked to select their preferred option. Options A and B coded alternative incentive schemes, while C described the option of no breastfeeding. Additionally, women were offered an opt-out option as a realistic alternative that they could choose if they wanted to breastfeed (and Option C was therefore not appealing to them), but neither Option A nor B could encourage them to do so.

We used a D-efficient design generated in Ngene (ChoiceMetrics Pty Ltd, Sydney, NSW, Australia), using zero priors and no restrictions, to identify the most efficient scenarios and choice set combinations to reduce the number of scenarios systematically while still being able to estimate main effects (i.e. direct independent effect of changes in one attribute on utility). While the maximum number of choice tasks per respondent was set as 8 to guarantee response efficiency, a statistically efficient design suggested a higher number of choice sets for a robust statistical analysis. Therefore, the final design included 24 choice sets that were divided into three versions of the questionnaire (see Table 1 for attributes, levels and level balance across the 24 choice sets). Participants were randomly allocated to one of the versions.

Ethical approval for this study was obtained from Brunel University Research Ethics Committee. Written informed consent was obtained from all participants.

### 2.2. Data

This study drew on the population-based South Yorkshire Cohort (SYC) [25] to identify women in IMD (Index of Multiple Deprivation) quintiles 3–5. Data were collected between January and March 2014. Out of 2,531 questionnaires posted to eligible women aged 16–45 years, 564 were returned following one mailed reminder. Following validity checks to ensure that respondents had read, understood and engaged constructively with the choice task, 2 questionnaires were excluded. For those respondents, the chosen options were found to be the same across all choice tasks indicating that respondents had not necessarily considered trade-offs between the presented scenarios. The useable response rate was therefore 22.2%. Each individual questionnaire provided a maximum of 8 choice responses, which resulted in a maximum of 4,496 possible choices. Since each choice included four options (A, B, C and opt-out) from which a respondent had to choose her preferred option, the maximum number of observations was 17,984. Five percent of choices were missing, which resulted in a final data set containing 17,084 observations.

### 2.3. Data analyses

Data were analysed based on a random utility framework. Conditional and mixed effects logistic regressions were fitted to estimate mean change in utility, value or preference, which respondents placed on an attribute level compared to the reference level. This assumes that the choices individuals make in a DCE reveal the utility they place on the alternatives presented. Following a utility maximisation framework, it is assumed that an individual will consider trade-offs between different attributes levels and choose an alternative in a given choice set if the utility derived from that alternative is greater than from any other alternative offered in the choice set [26].

The utility, U, derived from the alternative chosen is assumed to comprise of two parts: a systematic, observable component captured through the choices respondents make when answering DCE questions (in square brackets); and a stochastic, unobservable component, ε [27]. This can be expressed as:

$$U=\left[\alpha +\beta_{1}X_{1}+\beta_{2}X_{2}+\ldots +\beta_{n}X_{n}\right]+\varepsilon$$ (1)

where α is the alternative specific constant (ASC), X are attributes included in the DCE, β are the coefficients describing the marginal utility of that attribute, and ε is the unobservable component.

Effects coding was used for three attributes (breastfeeding duration, type of incentive, and method to verify breastfeeding) to allow the effects of each attribute to be uncorrelated with the constant and to calculate the effect of a reference category as the negative sum of the estimated coefficients for remaining attribute levels [28]. Coding of the opt-out remained the same across all choice sets. The ‘value’ attribute was assumed to be linear since the number of observations did not allow inclusion as categorical variable.

According to Eq (1), results are presented as coefficients in terms of ‘marginal utility values’ for each attribute level compared to the reference level. Marginal utility values indicate relative preferences for levels within an attribute, e.g. relative preference for vouchers for high street/local shops, or gift pack compared to direct cash transfer. Positive marginal utility values indicate an attribute level is preferred to the reference level and is associated with a positive preference (utility), while negative marginal utility values represent a negative preference (dis-utility) implying that the reference level is preferred over the specific attribute level.

The initial conditional (fixed effects) logit model assumed homogenous preferences across respondents. In order to account for the restricting assumptions of Model 1, we also applied mixed logit (MXL) models (Models 2 and 3) [29] with 1000 iterations. Rather than developing a priori assumptions, this more general approach allowed controlling for unobserved preference heterogeneity across the sample population [30]. For variables that entered the MXL model as random parameters, estimates are reported as mean effects and standard deviations, and as mean estimates only for fixed parameters. For both MXL models, the intercepts (i.e. alternative specific constants, ASCs) were assumed to be random and normally distributed (mean and standard deviations, SD, reported) while all other parameters remained fixed in Model 2 (only mean estimates reported). Additionally in Model 3, the attribute capturing breastfeeding duration was also assumed to be random, so that all random parameters in this model were the ASCs and two variables capturing different lengths of breastfeeding duration. Significant SDs for random parameters indicate that preference heterogeneity may exist and preferences for that specific attribute vary in the population. Preference heterogeneity can be present as difference in either the strength of the preference, i.e. the magnitude of the estimated coefficient, or the direction of preference, i.e. the sign of the estimated coefficient [30]. Finally, in order to investigate any underlying preference heterogeneity in greater detail, we included interaction terms between the ASCs and additional variables (age, children living in household, previous breastfeeding experience, intended breastfeeding duration, living with partner, IMD quintile) in a conditional logit model (Model 4). This allowed identifying which individual characteristics affected the underlying preferences and would therefore mediate the general preference for or against a financial incentive scheme while potentially controlling for some non-response bias in the results if characteristics between respondents and non-respondents differed significantly. Since we used effects coding for all attribute levels (which included negative values), interaction terms could only be included for the ASCs.

P-values are reported to describe the level of statistical significance for each marginal utility value.

Model goodness of fit is reported as likelihood ratios (LR). All analyses were run in STATA12 (StataCorp. 2012).

## 3. Results

### 3.1. Sample characteristics

Based on data available from the SYC, characteristics of respondents and non-respondents are described in Table 2. Respondents were on average older than non-respondents, more likely to have children and from a less deprived background.

**Table 2:** Descriptive statistics (SYC): Respondents vs. non-respondents.

	Summary statistics	
Variables	Respondents	Non-respondents
n	562	1971
	Mean (SD) / n (%)	Mean (SD) / n (%)
Age		
Mean age [years]	33.04 (6.88)	30.51 (7.80)***
16–24 years	67 (12)	523 (27)***
25–34 years	232 (41)	744 (38)
35–44 years	261 (46)	704 (36)***
Missing	2 (<1)	0 (0)
Children		
Yes	310 (55)	913 (46)***
No	200 (36)	769 (39)
Missing	52 (9)	289 (15)
IMD quintile		
3	199 (35)	527 (27)***
4	143 (25)	539 (27)
5 (most deprived)	218 (39)	905 (46)***
Missing	2 (<1)	0 (0)

*** statistically significantly different from ‘respondents’ at 1% level.

Additional characteristics of respondents based on self-reported information provided in the questionnaire are reported in Table 3. Around two thirds reported to have children (69%) and to be living with a partner (74%). The majority was employed or on maternity leave (81%) and 41% reported a weekly household income (including benefits) of more than £500/week.

**Table 3:** Sample characteristics (DCE).

Variables		Summary statistics	
	n	%	Mean (SD)
Number of returned questionnaires	562		
Version 1	191	34	
Version 2	174	31	
Version 3	197	35	
Chosen option (out of a maximum of 4,496 possible choices)	4,496		
A or B	3,413	76	
C (no breastfeeding)	216	5	
Opt-out	642	14	
Missing	210	5	
Part 1: Children and infant feeding	N = 562		
Children			
Yes	395	70	
No	167	30	
Number of children			
0	167	30	
1	150	27	
2	160	28	
3	42	7	
4	15	3	
5	5	1	
6	1	<1	
Missing	22	4	
Children: 0–2 months	16		1 (0)
Children: 3–6 months	21		1 (0)
Children: 7 months—2 years	99		1.09 (0.29)
Children: 3–5 years	107		1.08 (0.28)
Children: 6–12 years	164		1.32 (0.54)
Children: 13–18 years	103		1.31 (0.52)
Children: >18 years	45		1.38 (0.64)
Breastfed as child			
Yes	264	47	
No	219	39	
Don’t know	62	11	
Missing	17	3	
Breastfeeding experience (previous/current)			
Yes	342	61	
No	206	37	
Missing	14	2	
Longest breastfeeding duration			
1–6 days	46	13	
1–4 weeks	40	12	
1–2 months	34	10	
3–6 months	70	20	
>6 months	144	42	
Missing	8	2	
Pregnant			
Yes	34	6	
No	514	91	
Don’t know	1	<1	
Prefer not to say	1	<1	
Missing	12	2	
If pregnant: Plan to feed baby during first 6–8 weeks			
Not decided yet	6	18	
Breast milk only	17	50	
Formula only	2	6	
Breast milk & formula	7	21	
Missing	2	6	
Part 2: Your preferences (ranking exercises) (1—most preferred, 3 or 4—least preferred)			
Average rank: method of verifying breastfeeding			
Signed statement: mother			2.28 (0.87)
Signed statement: healthcare provider			2.00 (0.68)
Signed statement: both			1.71 (0.78)
Average rank: type of incentive			
Direct cash transfer			1.34 (1.34)
Vouchers: high street shops			2.10 (0.84)
Vouchers: local shops			2.56 (0.98)
Gift pack			2.94 (1.11)
Part 3: Choices			
Preferred breastfeeding duration			
6 weeks	71	13	
3 months	88	16	
6 months	307	55	
None of these	15	3	
Missing	81	14	
Part 4: Household characteristics			
Living with partner			
Yes	418	74	
No	123	22	
Prefer not to say	6	1	
Missing	15	3	
Employed/Maternity leave			
Yes	457	81	
No	91	16	
Missing	14	2	
Household income (including benefits)			
<100 £/week	13	2	
100-<200 £/week	33	6	
200-<300 £/week	53	9	
300-<400 £/week	72	13	
400-<500 £/week	64	11	
>500 £/week	232	41	
Don’t know	22	4	
Prefer not to say	57	10	
Missing	16	3	
Ever received ‘Healthy Start’ vouchers			
Yes	54	10	
No	491	87	
Missing	17	3	

Respondents reported on two types of breastfeeding; whether they had been breastfed as a child (47% were) and whether they had breastfed a child (61%). Out of those who had breastfed before, 12% reported their longest period of breastfeeding (not necessarily exclusively) as between 1–4 weeks and 20% for between 3–6 months, with 42% having at least one experience of continuing for more than 6 months. 10% of the whole sample had previously participated in a national incentive-based infant feeding (“Healthy Start’) scheme. Healthy Start is a statutory means-tested programme that provides vouchers (for fruits and vegetables, milk/infant formula) and coupons (for free vitamin supplements) to women who are at least 10 weeks pregnant and families of children who are up to four years old [31]. Women and families are eligible if they: (a) receive welfare benefits, (b) receive qualifying tax credits and their annual household income is £16,910 or less, (c) universal credit and family take home monthly pay of £408 or less. Women aged under 18 years and pregnant qualify regardless.

Six percent of the sample were currently pregnant. Out of those, 50% had already decided to breastfeed exclusively, 6% to bottle feed exclusively while for 18% it was unclear and the rest were planning a mix (21%).

Differences in the reported characteristics between both data sources with regard to the proportion of respondents reporting to have children might be explained by the time lag between data collection points. Information on some individual characteristics was used from the SYC only to reduce the burden to respondents when completing the questionnaire and should therefore be only considered an approximation of true individual characteristics.

### 3.2 Ranking exercises

The ranking exercises revealed that a signed statement by both mother and health care provider to verify breastfeeding was most preferred by almost half of the sample (47%) and least preferred by 19% of the sample. A signed statement by mothers only was most often the least preferred option (53%) but most preferred by 27% of the sample. Signing by the health care provider alone was equally ranked by 22% as the most and least preferred option.

Direct cash transfer was given the top ranked position most often (42%) but also had a high proportion (33%) giving the lowest rank. The ‘gift pack’ was given the lowest rank most frequently (40%) and the highest rank least frequently (15%). Vouchers for high street shops had the fewest bottom ranks (5%) and second highest most preferred rank (24%).

When asked about their breastfeeding duration, if their preferred financial scheme was available, more than half of women (55%) in the sample reported that they would breastfeed for at least 6 months. The proportion of women preferring 6 weeks and 3 months was similar (13 and 16% respectively) while for 3% none of the specified durations seemed favourable.

### 3.3. Regression models

Table 3 shows that all versions of the questionnaire were returned in similar proportions (31–35%). The majority of respondents (76%) chose either option A or B. Table 4 shows the marginal effects of each attribute on utility for the conditional (Model 1 and Model 4) and the mixed effects logistic (MXL) models (Model 2 and Model 3).

**Table 4:** Results.

Attributes and levels	Marginal utility values			
	Conditional logit model (Model 1)	MXL (Model 2)	MXL (Model 3)	Conditional logit model–interactions (Model 4)
Alternative-specific constants (ASCs)				
Option A or B (AB)				
mean	1.9926***	9.4250***	9.6639***	-2.5913***
SD	n/a	7.1606***	7.3180***	n/a
Option C (‘no breastfeeding’)^#^	-3.1039	-10.5249	-10.9463	2.9182
Opt out				
mean	1.1114***	1.0999*	1.2823**	-0.3269
SD	n/a	6.8995***	6.7956***	n/a
Duration of breastfeeding				
2 days^#^	0.1460	0.1548	0.1703	0.1640
10 days				
mean	-0.2153***	-0.2109***	-0.1937***	-0.2289***
SD	n/a	n/a	0.0050	n/a
42 days (6 weeks)				
mean	0.0693**	0.0561**	0.0234	0.0649**
SD	n/a	n/a	-0.5495***	n/a
Maximum amount [£]				
Amount	0.0005***	0.0005***	0.0007***	0.0005***
Type of financial incentive				
Direct cash transfer^#^	0.0243	0.0101	0.0843	0.0265
Vouchers: high street shops	0.0041	0.0174	-0.0175	0.0265
Vouchers: local shops	-0.2158***	-0.2338***	-0.2651***	-0.2616***
Gift pack	0.1874***	0.2063***	0.1982***	0.2086***
Method of confirmation: signed statement from:				
Mother^#^	0.0048	0.0078	0.0316	0.0069
Healthcare provider	-0.0208	-0.0391	-0.0594*	-0.0289
Both	0.0160	0.0313	0.0277	0.0220
Interactions with ASCs				
Age				
AB				-0.0395**
Opt-out				-0.0041
Number of children (compared to ‘no children’)				
1 child				
AB				-1.4422***
Opt-out				-1.8516***
2+ children				
AB				-2.1017***
Opt-out				-2.3030***
Previous breastfeeding experience				
AB				1.1832***
Opt-out				1.4596***
Intended breastfeeding duration (compared to ‘none of these durations’)				
≥6 weeks				
AB				5.1504***
Opt-out				0.8720***
≥3 months				
AB				5.4731***
Opt-out				1.0560***
≥6 months				
AB				6.5199***
Opt-out				2.0454***
Living with partner				
AB				0.7961***
Opt-out				0.8253***
IMD quintile (compared to 3^rd^ quintile)				
4^th^ quintile				
AB				1.7197***
Opt-out				1.4638***
5^th^ quintile				
AB				1.9448***
Opt-out				1.4367***

^#^ Reference category.

***/**/* statistically significantly different from reference category at 1%/5%/10% level. n/a–estimation of SD in conditional logit models not applicable. Number of observations = 16,872. Model 1: Log-likelihood = -4855.23; LR chi^2^(10) = 1984.32. Model 2: Log-likelihood = -3392.83; LR chi^2^(2) = 2924.81. Model 3: Log-likelihood = -3341.11; LR chi^2^(4) = 3028.24. Model 4: Log-likelihood = -3476.57; LR chi^2^(30) = 2531.88.

While results were found to be very similar across all three models, the MXL model that considered both the intercepts (ASCs) and the duration levels as random parameters (Model 3) was found to best fit the data based on LR tests. The results suggest that coefficients for most attribute levels are statistically significant and therefore the attribute level may have an impact on the probability of choosing an alternative.

Significant positive coefficients for the ASCs combined for options A and B, and the opt-out indicate that women on average preferred to breastfeed and participate in the scheme compared to option C ‘no breastfeeding’. Compared with a breastfeeding duration of 2 days, continuous breastfeeding for 10 days was associated with reduced utility while a duration of 6 weeks was preferred. However, the results suggest that preferences for the level of 6-week duration varied within the study population (Model 3). As expected, respondents had a strong preference to receive higher amounts. The effect of a one-pound increase in the average amount received was even larger when accounting for preference heterogeneity in Model 3. There was no statistically significant preference for high street vouchers compared to cash transfers. However, vouchers for local shops were less preferred, and a gift pack more preferred to cash. No evidence was found for differences in preferences for the specific verification methods.

Adding interactions between ASCs and additional variables (Model 4) improved the goodness of fit of the conditional logit model, but goodness of fit remained higher for both MXL models (Model 2 and 3). In Model 4, the significance or direction of marginal utility values for attribute levels did not change. However, when controlling for individual characteristics (age, number of children living in household, previous breastfeeding experience, intended breastfeeding duration, living with partner, IMD quintile) the results suggest that individual characteristics can mediate women’s preferences for a financial incentive scheme. The marginal utility value for option A or B remained significant, but was associated with a negative preference. No significant difference could be observed between the opt-out and the reference option of ‘no breastfeeding’. This implies that once individual and socio-economic characteristics are controlled for, the general preference for a financial incentive scheme can no longer be observed; respondents associated a disutility from participating in an incentive scheme compared with not breastfeeding at all (or not participating in an incentive scheme). Additionally, a negative coefficient for the age variable suggests that the older respondents were, the more disutility they experienced with choosing options A or B. If respondents had children, they also experienced additional disutility from choosing any incentive scheme, i.e. options A or B or the opt-out, compared with respondents without children. The disutility was even greater if women reported to have more than one child. However, all other characteristics controlled for in the analysis were associated with a gain in utility from choosing an incentive scheme: If women were living with their partner, had breastfed before or intended to breastfeed for any duration longer than 6 weeks, and if women were from IMD quintiles 4 and 5 (compared to IMD quintile 3), they preferred the incentive scheme to the option of no breastfeeding.

## 4. Discussion

### Summary of findings

This is the first DCE that has investigated women’s preferences on financial incentives for breastfeeding. Alternative scheme designs presented to participants in the DCE were described by different specifications of four attributes: maximum value of the incentive, minimum breastfeeding duration required to receive inventive payment, method of verifying breastfeeding, and type of incentive. We found evidence that most of the included attributes affect a woman’s probability to participate in the incentive scheme. On average, women preferred to breastfeed to participate in the scheme compared to no breastfeeding. There was a strong positive preference for higher values paid as an incentive. Participants considered cash transfers and high street vouchers equally preferable, while gift pack was more and vouchers for local shops were less preferred compared to cash transfer. On average, participants preferred longer breastfeeding duration of 6 weeks rather than 2 days, while a breastfeeding duration of 10 days was least preferred. However, preferences for breastfeeding durations were found to vary with individual characteristics of the study population. There was no evidence that using either or both mother’s or health professional’s signature to verify breastfeeding had an effect on individual utility and these are therefore not expected to influence mothers’ decisions to participate in the scheme significantly.

Individual characteristics and circumstances were found to affect women’s preferences for or against an incentive scheme significantly. Once individual characteristics were accounted for, financial incentives for breastfeeding were no longer preferred or desirable.

### Strengths & limitations

In absence of observational data, where an intervention has not been implemented yet and no evidence is available on its acceptability among potential recipients, stakeholder views are the best measure of acceptability available. Giles, Becker [6] found that financial incentives could be more socially acceptable and therefore effective in promoting healthy behaviour than previously assumed. Our results can improve the evidence around the acceptability of a financial incentive scheme for breastfeeding and help to estimate the effectiveness of the intervention when incentivising breastfeeding behaviour.

Since DCEs can be difficult for participants to understand, substantial pre-testing was conducted to minimise the risks and align participants understanding of the questionnaire with research aims. Furthermore, results are plausible and confirm the qualitative work including focus group interviews during the development stage [32]. We included an opt-out option to offer a realistic choice scenario and increase the probability that respondents engaged constructively with the task. We excluded responses from participants whose responses did not show any variation across choice tasks in order to reduce bias.

However, some of the findings need to be interpreted with caution. The generalisability of our findings may be limited by almost 55% of the study population indicating that they would breastfeed for at least 6 months and that fact that 42% had at least one experience of continuing for more than 6 months. Compared with a UK average of 34% for breastfeeding rates at 6 months [4], 42% of respondents in our sample with previous breastfeeding experience had at least on one occasion breastfed for more than 6 months. However, the inclusion of a variable for intended breastfeeding duration controlled for some selection bias in the results. The general preference for an incentive scheme changed into a disutility while the utilities for attribute levels were not affected at all. Watson, Becker [33] found that the relevance of the survey topic to respondents is positively associated with response rate, which may explain the proportion of the sample population with children that was above the UK national average. Compared to the general population respondents showed a preference towards breastfeeding in the survey, which may have influenced their opinion about a financial reward for breastfeeding and resulted in biased estimates.

However, the final analysis (Model 4) accounted for individual characteristics that were assumed to be correlated with a women’s alleged acceptability of an incentive scheme. Having children was found to be associated with a decrease in utility, which was even greater if more than one child was living in the household. This finding might be associated with a general time constraint, where the available time has to be divided across care of several children. Breastfeeding one child would require an additional time commitment from the mother and would decrease her time available for any other activity. Results from the statistical analyses provided further evidence that preferences varied in the study population depending on individual characteristics. Depending on those characteristics, specific groups would need to be identified to target those most at risk, e.g. from more deprived areas, to increase uptake of an incentive scheme and to enable a more effective delivery of the intervention.

Coverage, sampling, non-response and measurement errors are common limitations of DCE surveys [34]. While most DCE research focusses on minimising measurement error (e.g. [35–37]), research on non-response bias in health-related DCEs is lacking [33]. The response rate to our DCE was relatively low and may have led to non-response bias in the estimates. Due to limited guidance in the current literature, further considerations focusing on testing and accounting for non-response bias in DCE data are needed. However, response rates to DCEs, especially postal surveys, have been shown to decrease over time [33], while response rates to online surveys are usually not reported at all. Given the number of women initially approached, our sample size of 562 respondents was sufficiently large to run robust statistical analyses and inclusion of interaction terms with individual characteristics may have controlled for some of the assumed non-response bias. Non-respondents were on average younger, less likely to have children, and from a more deprived background, with all of these characteristics likely to be correlated. Controlling for these factors where differences between respondents and non-respondents were observable, should have reduced any potential non-response bias as far as possible given the available data.

### Interpretation of findings

Research around behavioural intentions has rarely involved the use of DCEs and has mainly aimed at smoking cessation [6, 38]. Giles, Becker [6] and Morgan, Hoddinott [39] found that incentives can be an effective measure to increase the likelihood of engaging in healthy behaviours, but that the relative effect of other characteristics of a scheme may be greater than the value of the incentive itself.

We found strong preferences for and distribution of responses to options A and B, which implies that, ceteris paribus, respondents were on average more likely to participate in an incentive scheme than not. However, once individual characteristics were included, the preference for financial incentives was reversed and women expressed a dislike for participation. Despite this change in general preference for/against an incentive scheme, preferences for different attributes describing the scheme did not change. While there was no evidence of preferences for the specific types of behaviour monitoring for receipt of vouchers, the effect of different types of incentives needs further consideration. Responses to the ranking exercise suggested that direct cash transfer was the most preferred, but also the least preferred for a substantial proportion of women (33%), while a gift pack was the least preferred option (40%) with the lowest average rank (Table 5). However, these results need to be considered with caution and do not provide guidance on preferences for/against an incentive scheme, since the ranking exercises would only consider each DCE attribute alone and not in combination with other characteristics of an incentive scheme.

**Table 5:** Ranking exercises–respondents [%] ranking types of benefits.

	Direct cash transfer	Vouchers: high street shops	Vouchers: local shops	Gift pack
Rank 1 (most preferred)	42	24	16	15
Rank 2	11	40	26	15
Rank 3	9	25	34	24
Rank 4 (least preferred)	33	5	17	40
Mean (SD) ranked position	2.34 (1.34)	2.10 (0.84)	2.56 (0.98)	2.94 (1.11)

When investigating preferences for the type of incentive in the DCE setting, i.e. in combination with the other attributes describing the scheme and therefore controlling for trade-offs between different characteristics women were willing to make, estimates from the regression models suggest that a gift pack was found to have a positive impact on utility compared to using direct cash transfer. An explanation for this observation may be that women found it easy to answer the ranking exercises from their own point of view (as they were asked to do) indicating that a more flexible reward would the more useful. In contrast, answers to the presented choice sets may have been influenced by what they thought best for other women in that situation, preferred for others or thought was necessary for others in order to ensure the rightful receipt of incentives. Although the question clearly stated to make choices from her own point of view, it was not possible to distinguish the respondent’s perspective from answers to the questionnaire, i.e. whether she answered from a personal or socially desirable perspective. Additionally, since the hypothetical scenario to answer all questions from a personal point of view was only described before the first choice set (see Fig 1), respondents’ perspective when completing the choice tasks could have moved away from the personal perspective while completing more and more choices. However, this issue had not been picked up during the pre-testing phase, although it had become obvious that a social desirability bias may occur since women thought it inappropriate for themselves to accept financial incentives to breastfeed. However, some indicated that it may be helpful for other women to have more funds available to spend on breastfeeding related items. Concerns were expressed that ‘other people’ could (mis-)use more flexible incentives such as cash transfers for unrelated items, which may explain the positive preference for a gift pack which would prevent any mis-use of additionally provided income. Alternatively, the alleged discrepancy in results for the gift pack option might be based on conceptual differences between the ranking exercises and the DCE choice sets. Women might not favour a gift pack when comparing it only to different types of incentives rather than to several other characteristics of a more complex incentive scheme. The preference in the DCE results might suggest that women find it easier to trade-off cash incentives or vouchers with a specific value for other characteristics of the incentive scheme, while the concept of a gift pack might be more abstract and therefore more difficult to trade-off in choice situations, resulting in a positive coefficient for this type. No significant preferences for any type of verification method might suggest that respondents did not consider any control necessary to confirm a change in behaviour.

### Implications of findings for policy, practice and research

Our findings show that strong preferences for and against specific characteristics of a financial incentive scheme exist. They indicate that financial incentives could be an effective intervention to promote breastfeeding and longer breastfeeding durations. The stronger a positive preference is, the lower the value of an incentive has to be that is required for a behaviour change. Similarly, identifying the least preferred characteristics of an incentive scheme may provide valuable information for effectiveness calculations.

In order for an intervention to be most effective, specific target groups would need to be identified according to key characteristics that would maximise their response in terms of behaviour change to a financial incentive. Evidence exists that financial incentives for health behaviours are socially more acceptable when targeted at vulnerable groups such as individuals living in deprived areas or low income circumstances [15, 40].

Giles, Becker [6] found evidence that the preferences for and acceptability of financial incentives varies with both the health behaviour they are aimed to improve and individual characteristics. Not accounting for individual characteristics results in biased estimates since the strength and direction of preferences within different groups of respondents may vary and may even cancel each other out. Our findings suggest that the use of financial incentives for breastfeeding might be more acceptable and therefore more effective in younger women from more deprived backgrounds. While previous breastfeeding experience suggested a preference for an incentive scheme, it would also be correlated with the presence of other children, who might reduce a mother’s time available to breastfeed. Future work should explore the reasons for some of the differences in preferences reported here and possibly address some of the methodological limitations. Combining the results from our survey with qualitative methods could help in distinguishing between preferences for incentives from personal and socially desirable perspectives, and inform the development of future trials investigating the effectiveness of incentive schemes.

## 5. Conclusions

Although public opinion might be mixed, financial incentives for breastfeeding could be an effective intervention. However, the type of incentive may substantially influence social acceptability of an incentive scheme in general as well as a woman’s likelihood to breastfeed. While women might be more willing to trade off cash incentives or shopping vouchers against other scheme characteristics, the preference for ‘gift pack’ may be based on women’s understanding of how an incentive should be used since it is less easily misused and therefore its potential for trade-offs might be considered limited. Rather than providing more flexible incentives that could be used at the recipient’s discretion, some control over the use of the additional income may have been preferred, while no confirmation of a behaviour change was found to be expected. Further work will be required to analyse how individual characteristics may influence preferences and to identify specific target groups who would benefit most from financial incentives for breastfeeding.

## Supporting information

S1 Questionnaire (PDF)
