# Supplementary material for: Women’s preferences for alternative financial incentive schemes for breastfeeding: A discrete choice experiment
Source: PLoS One. 2018 Apr 12;13(4):e0194231. doi: 10.1371/journal.pone.0194231 (PMC5896913; doi:10.1371/journal.pone.0194231)
Supplement: S1 Questionnaire — (PDF) [file pone.0194231.s001.pdf]

Questionnaire on a possible breastfeeding scheme:

Your preferences and views

Breastfeeding can improve the health of both mothers and babies. Breast milk can provide babies with all the food they need for the first 6 months of their life. It can help to protect them from infections and other diseases. It can also reduce the mothers' risks of developing future illnesses.

Because of these benefits, researchers are looking at a number of ways to promote breastfeeding, especially in areas where breastfeeding rates are very low.

This questionnaire asks you to think about a scheme that could give shopping vouchers, money or gift packs to mums who breastfeed or give breast milk to their babies. The scheme could be set up in different ways and we would like to know which you would prefer, if any. We will keep any information you give strictly confidential.

Everybody's experience and expectations of breastfeeding are different. There are no 'right' or 'wrong' answers. It is **your** views that are important to us, and that will help in designing an effective scheme.

The questionnaire takes around 10-15 minutes to complete.

If you have any questions about this questionnaire, please contact Ailish Higgins (call 01895 266902 or email ailish.higgins@brunel.ac.uk)

Thank you for your time in completing this questionnaire.

Please return the questionnaire in the FREEPOST envelope (no stamp required).

Brunel UNIVERSITY LONDON

The University Of Sheffield.

Part 1: Children and infant feeding

Q1. Do you have any children?

Please tick ONE box only.

- ☐ Yes
- ☐ No → Go to **Q3**

Q2. Please indicate how many children you have in each age group below:

	0-2 months
	3-6 months
	7 months -2 years
	3-5 years
	6-12 years
	13-18 years
	Older than 18 years

Q3. Were you breastfed as a child?

Yes	No	Don't know	Prefer not to say
☐	☐	☐	☐

Q4. Have you breastfed before or are you currently breastfeeding?

- ☐ Yes
- ☐ No → Go to **Q6**

Q5. What is the longest you have breastfed a child for? (*tick one box only)*

1-6 days	1-4 weeks	5-8 weeks	3-6 months	More than 6 months
☐	☐	☐	☐	☐

Q6. Are you currently pregnant?

Please tick ONE box only.

- ☐ Yes
- ☐ No → Go to **Q8**
- ☐ Don't know → Go to **Q8**
- ☐ Prefer not to say → Go to **Q8**

Q7. Have you decided how you will feed your baby for the first 6-8 weeks?

Please tick ONE box only.

- ☐ No, I have not decided
- ☐ Yes - breast milk only
- ☐ Yes - formula only
- ☐ Yes - both breast and formula milk

Part 2: Your preferences

In this section, we are interested in your views about some specific parts of a scheme to promote breastfeeding.

In order to qualify for the scheme, the organisers will need to confirm whether mothers are breastfeeding (or giving their baby breast milk).

Q8. Please rank each method of confirming breastfeeding below in your order of preference from 1-3:

	1 to 3
Signed statement from you that your baby is being fed breast milk	
Signed statement by a healthcare provider (a midwife, health visitor or breastfeeding peer support worker)	
Signed statement by you and a health care provider	

1=most preferred
3=least preferred

Please use each number only once.

Q9. Please rank the following in your order of preference from 1-4:

	1 to 4
Direct cash transfer to bank or post office account	
Range of vouchers for *high street* shops, like ASDA, Boots and WHSmith	
Range of vouchers for *local* shops and services, like local hairdressers, bakeries and fruit and vegetable shops	
A gift pack with pamper items, healthy treats and magazines	

1=most preferred
4=least preferred

Please use each number only once.

Part 3: Making choices

In the next part, we ask you to imagine that you are going to have a baby and that a new scheme encouraging you to breastfeed or give breast milk is running in your area.

To be eligible for the scheme when your baby is born, you would need;

- ***to breastfeed or give breast milk to your baby, and***
- ***to provide confirmation of breast milk feeding up to three times (when your baby is 2 days, 10 days, and 6 weeks old).***

There are eight choices asking you to choose between three options. Each option differs in:

- *whether or not money, high street (e.g. Asda, Boots) or local shopping vouchers (e.g. local hairdressers, baker), or a gift pack (e.g. with pamper items, magazine) is received;*
- *the method of confirming breastfeeding, if any;*
- *the maximum total value;*
- *how long breastfeeding would need to last for in order to qualify for the scheme.*

Please read all of the choices carefully. "Option C" is always the same - a situation where you do not breastfeed.

Please indicate, by ticking only ONE box from ***each*** *choice set, which option you would choose. In each choice set, you can select "None of the above" if you would not choose any of the options offered. For each choice set, assume that nothing else differs between options.*

There are no right or wrong answers. We are interested in YOUR views.

Imagine you were going to have a baby. Which option would you choose?

**Choice 1**	**Option A**	**Option B**	**Option C**
Minimum length of time you need to breastfeed for after birth	2 days	10 days	0 days
Maximum total value	£120	£80	£0
What you receive	Vouchers for *local* shops	Direct cash transfer	None
Method to confirm breastfeeding	Your signature	Healthcare provider's signature	Not needed

Which option would you choose? (*tick one box only)*

☐ Option A ☐ Option B ☐ Option C ☐ None of the above

**Choice 2**	**Option A**	**Option B**	**Option C**
Minimum length of time you need to breastfeed for after birth	10 days	6 weeks	0 days
Maximum total value	£80	£20	£0
What you receive	Gift pack	Vouchers for *local* shops	None
Method to confirm breastfeeding	Your signature	Healthcare provider's signature	Not needed

Which option would you choose? (*tick one box only)*

☐ Option A ☐ Option B ☐ Option C ☐ None of the above

**Choice 3**	**Option A**	**Option B**	**Option C**
Minimum length of time you need to breastfeed for after birth	6 weeks	2 days	0 days
Maximum total value	£20	£600	£0
What you receive	Direct cash transfer	Vouchers for *high street* shops	None
Method to confirm breastfeeding	Healthcare provider's signature	Healthcare provider and your signatures	Not needed

Which option would you choose? (*tick one box only)*

☐ Option A ☐ Option B ☐ Option C ☐ None of the above

Choice 4	Option A	Option B	Option C
Minimum length of time you need to breastfeed for after birth	10 days	2 days	0 days
Maximum total value	£240	£120	£0
What you receive	Gift pack	Vouchers for *high street* shops	None
Method to confirm breastfeeding	Healthcare provider and your signatures	Your signature	Not needed

Which option would you choose? (*tick one box only)*

☐ Option A ☐ Option B ☐ Option C ☐ None of the above

Choice 5	Option A	Option B	Option C
Minimum length of time you need to breastfeed for after birth	2 days	10 days	0 days
Maximum total value	£20	£600	£0
What you receive	Gift pack	Direct cash transfer	None
Method to confirm breastfeeding	Healthcare provider and your signatures	Your signature	Not needed

Which option would you choose? (*tick one box only)*

☐ Option A ☐ Option B ☐ Option C ☐ None of the above

Choice 6	Option A	Option B	Option C
Minimum length of time you need to breastfeed for after birth	10 days	6 weeks	0 days
Maximum total value	£240	£120	£0
What you receive	Vouchers for *local* shops	Vouchers for *high street* shops	None
Method to confirm breastfeeding	Healthcare provider's signature	Healthcare provider and your signatures	Not needed

Which option would you choose? (*tick one box only)*

☐ Option A ☐ Option B ☐ Option C ☐ None of the above

**Choice 7**	**Option A**	**Option B**	**Option C**
Minimum length of time you need to breastfeed for after birth	6 weeks	10 days	0 days
Maximum total value	£120	£20	£0
What you receive	Vouchers for *local* shops	Gift pack	None
Method to confirm breastfeeding	Healthcare provider's signature	Your signature	Not needed

Which option would you choose? (*tick one box only)*

☐ Option A ☐ Option B ☐ Option C ☐ None of the above

**Choice 8**	**Option A**	**Option B**	**Option C**
Minimum length of time you need to breastfeed for after birth	2 days	10 days	0 days
Maximum total value	£80	£120	£0
What you receive	Direct cash transfer	Gift pack	None
Method to confirm breastfeeding	Your signature	Healthcare provider and your signatures	Not needed

Which option would you choose? (*tick one box only)*

☐ Option A ☐ Option B ☐ Option C ☐ None of the above

Q10. If you were able to breastfeed, and the option you most preferred was available, would you breastfeed your baby for *at least*:

**a:**	6 weeks?	Yes ☐	No ☐	Don't know ☐
**b**:	3 months?	Yes ☐	No ☐	Don't know ☐
**c:**	6 months?	Yes ☐	No ☐	Don't know ☐

Part 4: A little more about you and your household

Nearly there! This last section asks questions that will help us understand more about your answers. Some may seem a little personal, but they are all useful. We promise to keep all of your information completely confidential.

Q11. Are you currently living with your partner?

Please tick ONE box only.

- ☐ Yes
- ☐ No
- ☐ Prefer not to say

Q12. Are you currently employed or on maternity leave?

Please tick ONE box only.

- ☐ Yes
- ☐ No

Q13. Which of these represents the income for your household from all sources of income (including benefits) before tax and national insurance?

Please tick ONE box only.

- ☐ Less than £100 per week
- ☐ £100 to less than £200 per week
- ☐ £200 to less than £300 per week
- ☐ £300 to less than £400 per week
- ☐ £400 to less than £500 per week
- ☐ More than £500 per week
- ☐ Don’t know
- ☐ Prefer not to say

Q14. Have you or your children ever received any Healthy Start vouchers that you can exchange for milk, formula, fruit, and/or vegetables?

Please tick ONE box only.

- ☐ Yes
- ☐ No

Q15. Is there anything else you would like to let us know about vouchers for breastfeeding? ____

____

____

____

Thank you very much for taking the time to complete this questionnaire
Please post it to us in the FREEPOST envelope provided.
